# 1-Naphthamide

**DOI:** 10.1107/S2414314626009892

**Published:** 2026-09-30

**Authors:** Mohamed Abdellatif Bensegueni, Rochdi Ghallab, Dorsaf Gheries, Asma Seghiri, Lamis Nouail

**Affiliations:** a Environmental Molecular and Structural Chemistry Research Unit University of Constantine-1 25000 Constantine Algeria; b Laboratoire de Technologie des Matériaux Avancés Ecole Nationale, Polytechnique, de Constantine Algeria; c CRM2 UMR CNRS 7036 Faculté des Sciences et Technologies Université de Lorraine, Institut Jean Barriol BP 70239 54506 Vandoeuvre-lès-Nancy Cedex France; University of Aberdeen, United Kingdom

**Keywords:** crystal structure, naphthalene ring, hydrogen bonds

## Abstract

In the title compound, C_11_H_9_NO, the dihedral angle between the naphthalene fused-ring system and the amide group is 42.18 (9)°. In the crystal, N—H⋯O hydrogen bonds link the mol­ecules into (100) sheets. No significant aromatic π–π stacking is observed.

## Abstract 2

In the title compound, the dihedral angle between the naphthalene fused-ring system and the amide group is 42.18 (9)°. In the crystal, N—H⋯O hydrogen bonds link the mol­ecules into (100) sheets.

## Graphical abstract

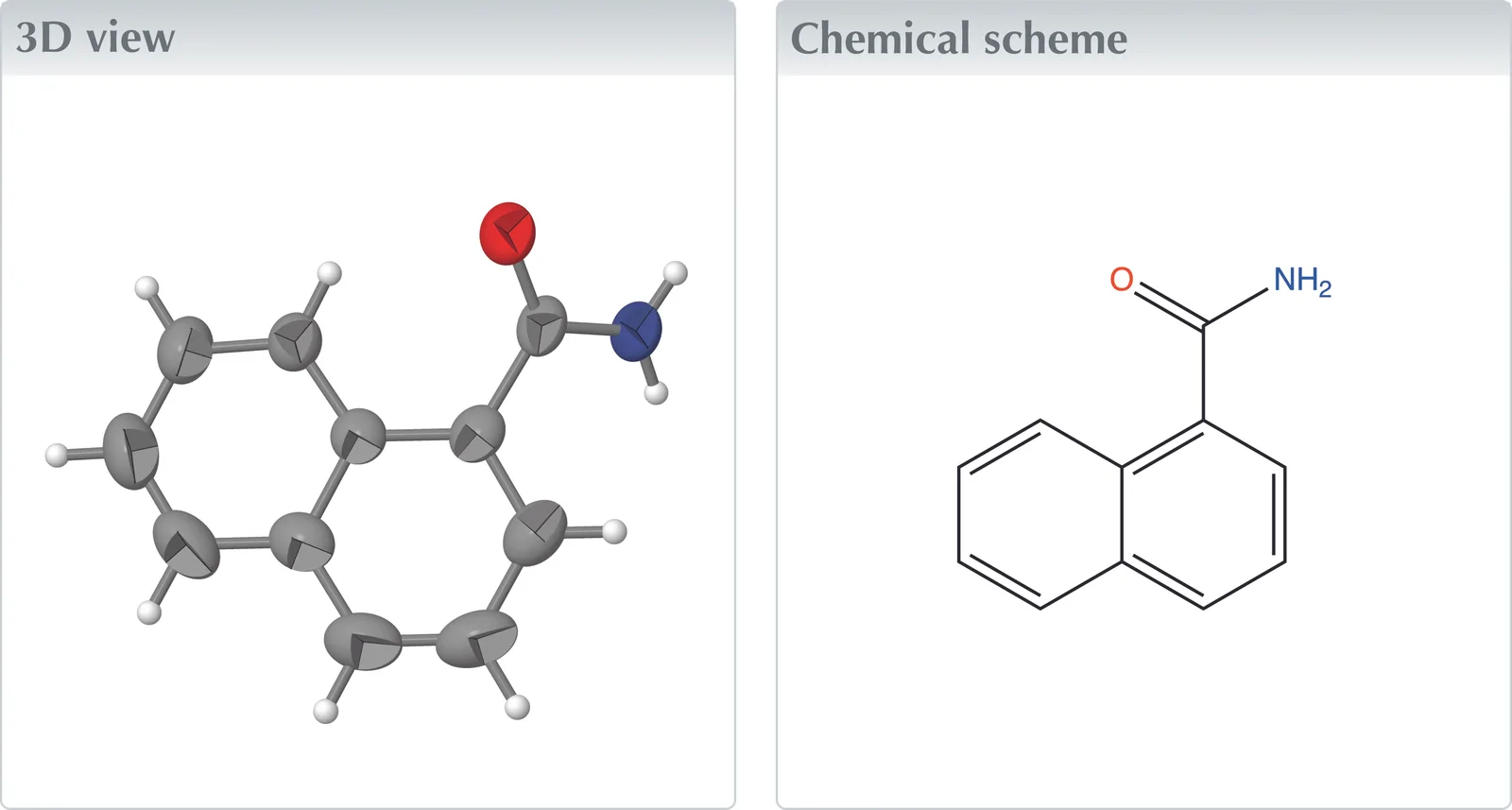

## Structure description

**Figure 1:**
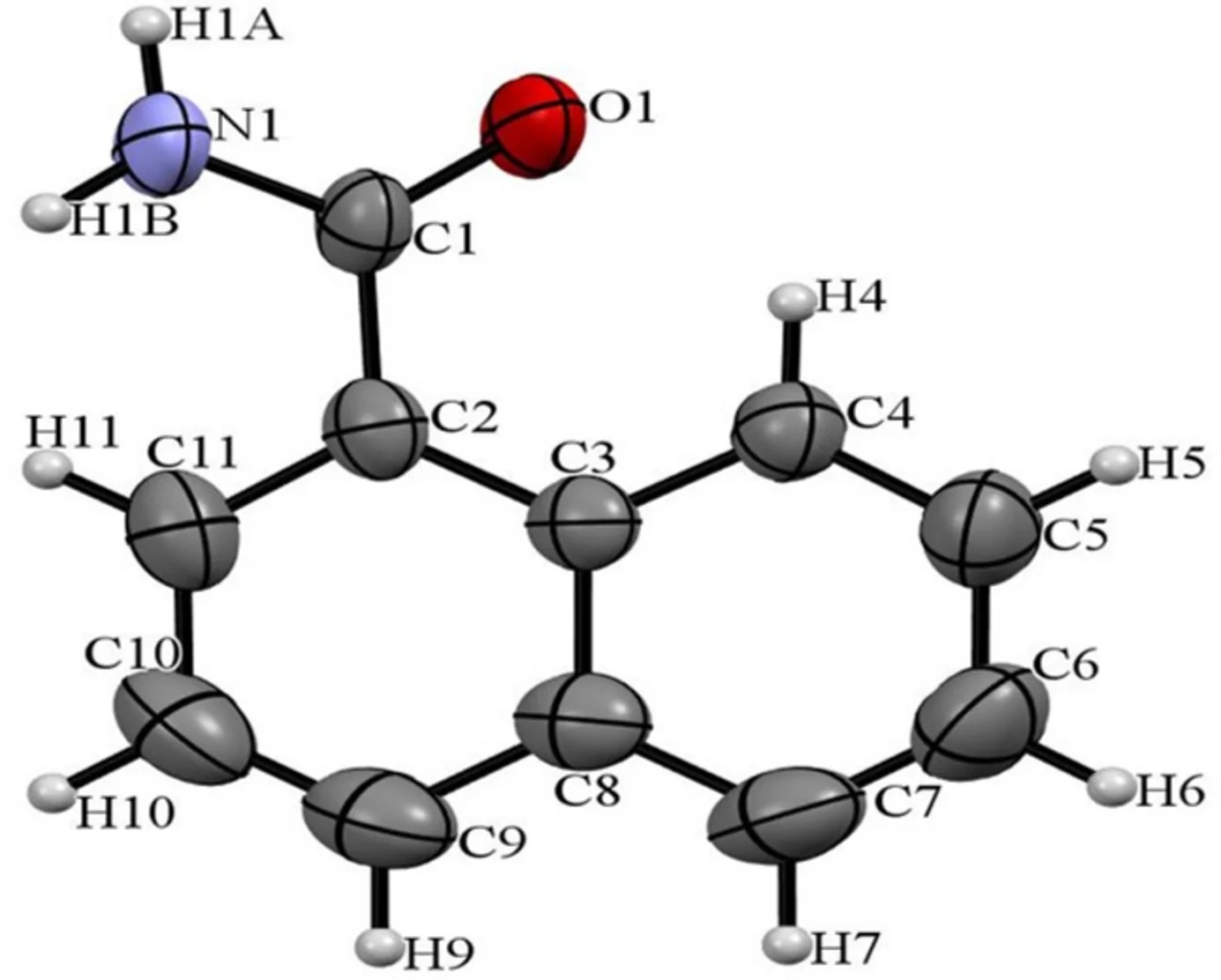
The mol­ecular structure of (**I**) with displacement ellipsoids drawn at the 50% probability level.

The title compound (**I**), with mol­ecular formula C_11_H_9_NO, crystallizes in the monoclinic space group *P*2_1_/*c*. The asymmetric unit contains one mol­ecule in which the dihedral angle between the C2–C11 fused ring system (r.m.s. deviation = 0.008 Å) and the C1/N1/O1 amide moiety is 42.18 (9)° (Fig. 1 ▸). The significant twist probably arises to minimize steric crowding. The geometrical parameters for the amide group [C1—N1 = 1.3249 (19) Å, C1—O1 = 1.245 (2) Å and angles O1—C1—N1, O1—C1—C2 and N1—C1—C2 close to 120°) support a conjugated amide system, as expected. A short intra­molecular C4—H4⋯O1 contact occurs.

**Figure 2:**
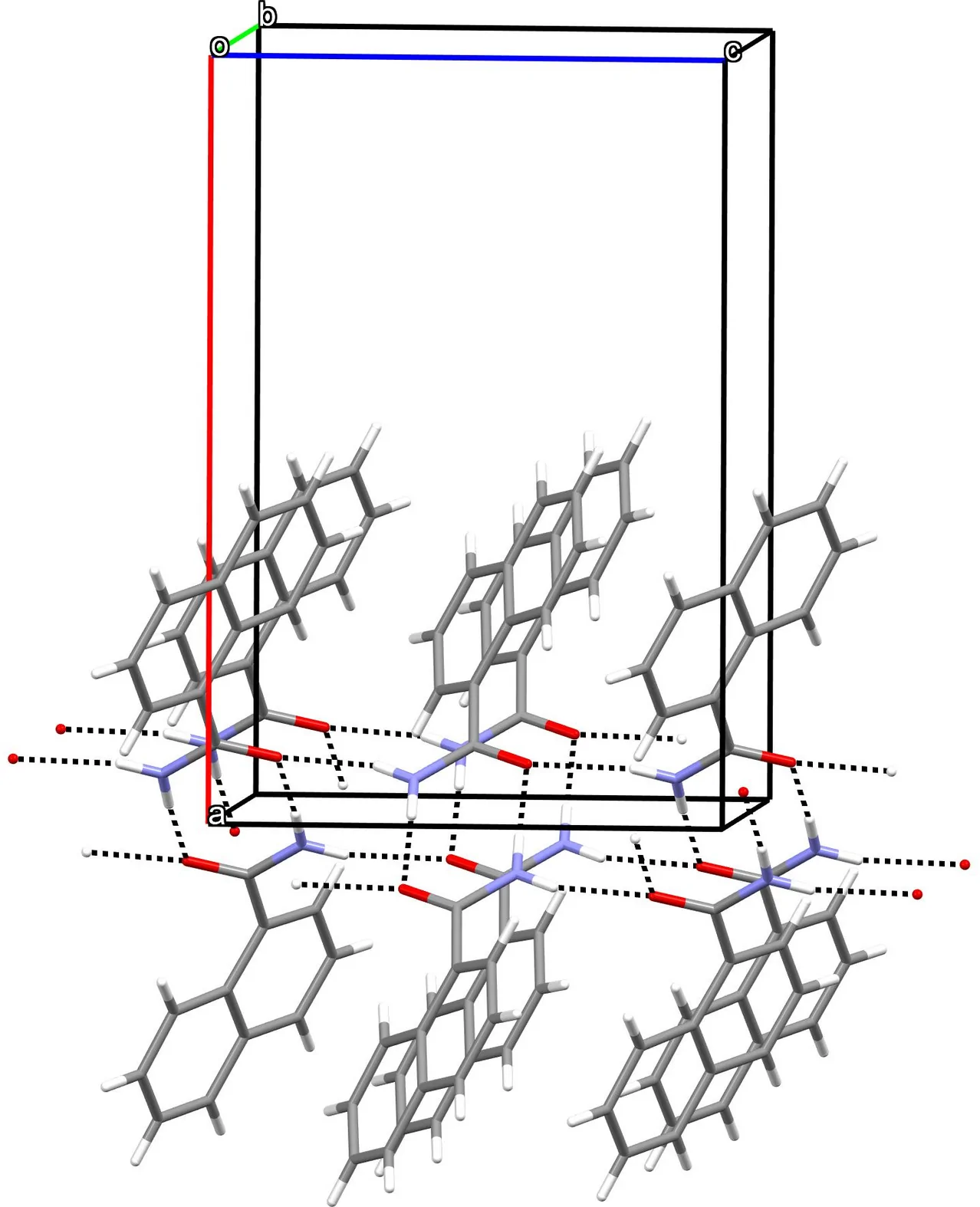
The packing in (**I**) with hydrogen bonds shown as black dashed lines.

In the extended structure of (**I**), the crystal cohesion is dominated by N11—H1*A*⋯O1 and N1—H1*B*⋯O1 hydrogen bonds (Table 1 ▸). The former generate inversion dimers and the latter link the dimers into (100) sheets (Fig. 2 ▸ and Table 1 ▸). The separations between the aromatic ring planes [shortest centroid–centroid separation = 4.799 (4) Å] are too large to support significant aromatic π–π stacking inter­actions.

**Figure 3:**
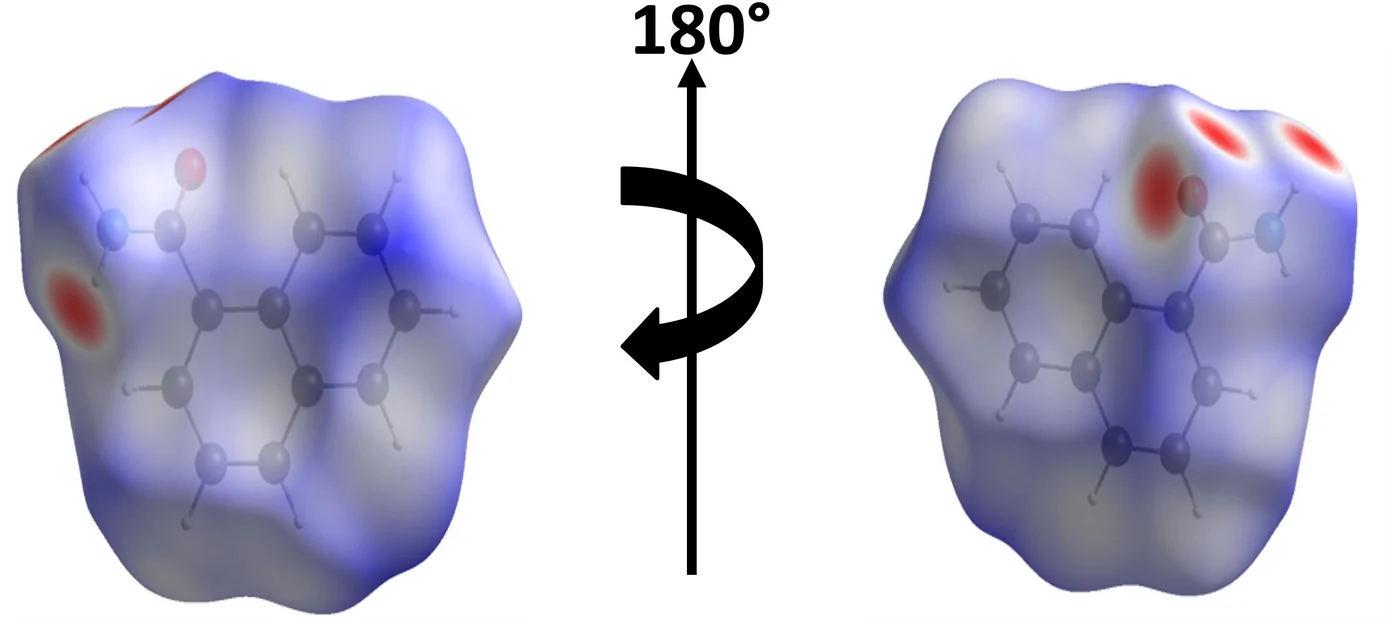
Hirshfeld surfaces of (**I**) mapped over *d*_norm_, shown as front and back views.

Hirshfeld surface analysis and the associated two-dimensional fingerprint plots were used to qu­antify the inter­molecular contacts in the crystal of (**I**) (Fig. 3 ▸). The fingerprint plots show that H⋯H contacts make the largest contribution (42.2%), followed closely by C⋯H/H⋯C contacts (40.2%). The O⋯H/H⋯O contacts account for 14.20%, while all other contributions are less than 2.0%. These results show that the cohesion of the crystal is primarily governed by van der Waals contacts, with aromatic π–π stacking inter­actions not playing a significant role in establishing the supra­molecular architecture.

For the related structure of 9-anthramide, see Heller & Schmidt (1971 ▸).

## Synthesis and crystallization

1-Naphthamide was obtained by aqueous hydrolysis of 1-naphtho­nitrile under heating. The starting nitrile was suspended in water and the mixture was heated until complete conversion to the amide was achieved. Colourless blocks of (**I**) were grown by slow evaporation of an aqueous solution of the compound at room temperature.

## Refinement

Crystal data, data collection and structure refinement details are summarized in Table 2 ▸.

## Supplementary Material

Crystal structure: contains datablock(s) I. DOI: 10.1107/S2414314626009892/hb4572sup1.cif

Structure factors: contains datablock(s) I. DOI: 10.1107/S2414314626009892/hb4572Isup2.hkl

Supporting information file. DOI: 10.1107/S2414314626009892/hb4572Isup3.cml

CCDC reference: 2590330

Additional supporting information:  crystallographic information; 3D view; checkCIF report

## Figures and Tables

**Table 1:** Hydrogen-bond geometry (Å, °)

*D*—H⋯*A*	*D*—H	H⋯*A*	*D*⋯*A*	*D*—H⋯*A*
C4—H4⋯O1	0.93	2.38	2.969 (3)	121
N1—H1*A*⋯O1^i^	0.86	2.10	2.951 (3)	170
N1—H1*B*⋯O1^ii^	0.86	2.06	2.889 (3)	163

Symmetry codes: (i) 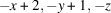; (ii) 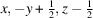.

**Table 2:** Experimental details

Crystal data	
Chemical formula	C_11_H_9_NO
*M*_r_	171.19
Crystal system, space group	Monoclinic, *P*2_1_/*c*
Temperature (K)	293
*a*, *b*, *c* (Å)	14.787 (5), 6.053 (5), 9.954 (5)
β (°)	90.448 (5)
*V* (Å^3^)	890.9 (9)
*Z*	4
Radiation type	Mo *K*α
μ (mm^−1^)	0.08
Crystal size (mm)	0.14 × 0.13 × 0.11

Data collection	
Diffractometer	Bruker APEXII
Absorption correction	Multi-scan (*CrysAlis PRO*; Rigaku OD, 2015 ▸)
*T*_min_, *T*_max_	0.753, 1.000
No. of measured, independent and observed [*I* > 2σ(*I*)] reflections	5594, 2584, 1535
*R*_int_	0.075
(sin θ/λ)_max_ (Å^−1^)	0.704

Refinement	
*R*[*F*^2^ > 2σ(*F*^2^)], *wR*(*F*^2^), *S*	0.071, 0.207, 1.05
No. of reflections	2584
No. of parameters	118
H-atom treatment	H-atom parameters constrained
Δρ_max_, Δρ_min_ (e Å^−3^)	0.22, −0.27

Computer programs: *CrysAlis PRO* (Rigaku OD, 2015 ▸), *OLEX2.solve* 1.5 (Bourhis *et al.*, 2015 ▸), *SHELXL* (Sheldrick, 2015 ▸) and *OLEX2* (Dolomanov *et al.*, 2009 ▸).

## full crystallographic data

### Naphthalene-1-carboxamide Crystal data

**Table:**

C_11_H_9_NO	*F*(000) = 360
*M**_r_* = 171.19	*D*_x_ = 1.276 Mg m^−^^3^
Monoclinic, *P*2_1_/*c*	Mo *K*α radiation, λ = 0.71073 Å
*a* = 14.787 (5) Å	Cell parameters from 2494 reflections
*b* = 6.053 (5) Å	θ = 2.5–35.6°
*c* = 9.954 (5) Å	µ = 0.08 mm^−^^1^
β = 90.448 (5)°	*T* = 293 K
*V* = 890.9 (9) Å^3^	Block, colourless
*Z* = 4	0.14 × 0.13 × 0.11 mm

### Naphthalene-1-carboxamide Data collection

**Table:**

Saxi-CrysAlisPro-abstract goniometer imported SAXI images diffractometer	2584 independent reflections
Radiation source: fine-focus sealed X-ray tube, Enhance (Mo) X-ray Source	1535 reflections with *I* > 2σ(*I*)
Graphite monochromator	*R*_int_ = 0.075
ω scans	θ_max_ = 30.0°, θ_min_ = 3.6°
Absorption correction: multi-scan (CrysAlisPro; Rigaku OD, 2015)	*h* = −9→20
*T*_min_ = 0.753, *T*_max_ = 1.000	*k* = −8→5
5594 measured reflections	*l* = −13→13

### Naphthalene-1-carboxamide Refinement

**Table:**

Refinement on *F*^2^	Primary atom site location: dual
Least-squares matrix: full	Hydrogen site location: inferred from neighbouring sites
*R*[*F*^2^ > 2σ(*F*^2^)] = 0.071	H-atom parameters constrained
*wR*(*F*^2^) = 0.207	*w* = 1/[σ^2^(*F*_o_^2^) + (0.106*P*)^2^ + 0.0779*P*] where *P* = (*F*_o_^2^ + 2*F*_c_^2^)/3
*S* = 1.05	(Δ/σ)_max_ < 0.001
2584 reflections	Δρ_max_ = 0.22 e Å^−^^3^
118 parameters	Δρ_min_ = −0.27 e Å^−^^3^
0 restraints	

### Naphthalene-1-carboxamide Special details

**Table:**

Geometry. All esds (except the esd in the dihedral angle between two l.s. planes) are estimated using the full covariance matrix. The cell esds are taken into account individually in the estimation of esds in distances, angles and torsion angles; correlations between esds in cell parameters are only used when they are defined by crystal symmetry. An approximate (isotropic) treatment of cell esds is used for estimating esds involving l.s. planes.
Refinement. The H atoms were placed geometrically (N—H = 0.86 Å, C—H = 0.93 Å) and refined as riding atoms.

### Naphthalene-1-carboxamide Fractional atomic coordinates and isotropic or equivalent isotropic displacement parameters (Å^2^)

**Table:**

	*x*	*y*	*z*	*U*_iso_*/*U*_eq_	
O1	0.92484 (9)	0.3228 (2)	0.10353 (11)	0.0589 (4)	
C11	0.84713 (15)	−0.1053 (3)	−0.10734 (18)	0.0600 (5)	
H11	0.9018	−0.1167	−0.1524	0.072*	
C2	0.83223 (12)	0.0721 (3)	−0.02377 (15)	0.0456 (4)	
C1	0.90457 (11)	0.2427 (3)	−0.00788 (15)	0.0472 (4)	
C3	0.74867 (11)	0.0887 (3)	0.04678 (15)	0.0439 (4)	
C4	0.72663 (12)	0.2652 (3)	0.13314 (17)	0.0509 (4)	
H4	0.7684	0.3780	0.1470	0.061*	
C8	0.68255 (13)	−0.0809 (3)	0.02734 (19)	0.0550 (5)	
C7	0.59888 (15)	−0.0640 (4)	0.0951 (2)	0.0721 (6)	
H7	0.5555	−0.1735	0.0827	0.086*	
C9	0.70104 (17)	−0.2580 (3)	−0.0600 (2)	0.0681 (6)	
H9	0.6579	−0.3681	−0.0725	0.082*	
C5	0.64530 (15)	0.2736 (4)	0.1967 (2)	0.0659 (6)	
H5	0.6326	0.3912	0.2537	0.079*	
C10	0.78034 (18)	−0.2706 (3)	−0.1257 (2)	0.0701 (6)	
H10	0.7912	−0.3884	−0.1833	0.084*	
C6	0.58088 (15)	0.1078 (5)	0.1774 (2)	0.0772 (7)	
H6	0.5256	0.1156	0.2210	0.093*	
N1	0.94732 (11)	0.3035 (3)	−0.11836 (13)	0.0605 (5)	
H1A	0.9900	0.3999	−0.1139	0.073*	
H1B	0.9324	0.2465	−0.1945	0.073*	

### Naphthalene-1-carboxamide Atomic displacement parameters (Å^2^)

**Table:**

	*U*^11^	*U*^22^	*U*^33^	*U*^12^	*U*^13^	*U*^23^
O1	0.0605 (8)	0.0885 (10)	0.0276 (6)	−0.0165 (7)	0.0030 (5)	−0.0033 (6)
C11	0.0731 (13)	0.0653 (12)	0.0416 (9)	0.0114 (10)	0.0017 (8)	−0.0031 (8)
C2	0.0543 (10)	0.0526 (10)	0.0299 (7)	0.0032 (7)	−0.0019 (6)	0.0028 (7)
C1	0.0478 (9)	0.0653 (11)	0.0285 (7)	0.0027 (7)	0.0025 (6)	0.0008 (7)
C3	0.0499 (9)	0.0482 (9)	0.0335 (7)	−0.0009 (7)	−0.0040 (6)	0.0066 (6)
C4	0.0527 (10)	0.0581 (10)	0.0418 (9)	−0.0015 (8)	0.0036 (7)	0.0006 (7)
C8	0.0630 (11)	0.0545 (10)	0.0472 (9)	−0.0073 (8)	−0.0089 (8)	0.0095 (8)
C7	0.0613 (13)	0.0844 (15)	0.0703 (14)	−0.0239 (11)	−0.0050 (10)	0.0150 (12)
C9	0.0880 (16)	0.0549 (12)	0.0612 (13)	−0.0146 (10)	−0.0138 (11)	0.0033 (9)
C5	0.0580 (11)	0.0825 (14)	0.0572 (12)	0.0048 (10)	0.0090 (9)	−0.0071 (10)
C10	0.1031 (18)	0.0500 (11)	0.0572 (12)	0.0029 (11)	−0.0113 (12)	−0.0088 (9)
C6	0.0538 (12)	0.1050 (18)	0.0730 (15)	−0.0059 (12)	0.0124 (10)	0.0030 (13)
N1	0.0655 (10)	0.0879 (11)	0.0281 (7)	−0.0179 (8)	0.0101 (6)	−0.0073 (7)

### Naphthalene-1-carboxamide Geometric parameters (Å, º)

**Table:**

O1—C1	1.245 (2)	C8—C9	1.408 (3)
C11—H11	0.9300	C7—H7	0.9300
C11—C2	1.377 (3)	C7—C6	1.352 (3)
C11—C10	1.417 (3)	C9—H9	0.9300
C2—C1	1.495 (3)	C9—C10	1.349 (3)
C2—C3	1.430 (2)	C5—H5	0.9300
C1—N1	1.3249 (19)	C5—C6	1.396 (3)
C3—C4	1.411 (2)	C10—H10	0.9300
C3—C8	1.430 (3)	C6—H6	0.9300
C4—H4	0.9300	N1—H1A	0.8600
C4—C5	1.364 (3)	N1—H1B	0.8600
C8—C7	1.417 (3)		
			
C2—C11—H11	119.5	C8—C7—H7	119.3
C2—C11—C10	120.9 (2)	C6—C7—C8	121.37 (19)
C10—C11—H11	119.5	C6—C7—H7	119.3
C11—C2—C1	119.01 (17)	C8—C9—H9	119.5
C11—C2—C3	119.67 (17)	C10—C9—C8	121.05 (19)
C3—C2—C1	121.32 (15)	C10—C9—H9	119.5
O1—C1—C2	122.04 (14)	C4—C5—H5	119.6
O1—C1—N1	121.23 (17)	C4—C5—C6	120.8 (2)
N1—C1—C2	116.73 (14)	C6—C5—H5	119.6
C2—C3—C8	118.39 (16)	C11—C10—H10	119.8
C4—C3—C2	123.88 (15)	C9—C10—C11	120.35 (19)
C4—C3—C8	117.72 (17)	C9—C10—H10	119.8
C3—C4—H4	119.3	C7—C6—C5	120.0 (2)
C5—C4—C3	121.30 (18)	C7—C6—H6	120.0
C5—C4—H4	119.3	C5—C6—H6	120.0
C7—C8—C3	118.83 (18)	C1—N1—H1A	120.0
C9—C8—C3	119.63 (19)	C1—N1—H1B	120.0
C9—C8—C7	121.54 (19)	H1A—N1—H1B	120.0
			
C11—C2—C1—O1	−137.42 (18)	C3—C8—C7—C6	−0.2 (3)
C11—C2—C1—N1	41.7 (2)	C3—C8—C9—C10	0.1 (3)
C11—C2—C3—C4	−179.15 (16)	C4—C3—C8—C7	0.0 (2)
C11—C2—C3—C8	−0.3 (2)	C4—C3—C8—C9	178.83 (16)
C2—C11—C10—C9	−0.7 (3)	C4—C5—C6—C7	0.2 (4)
C2—C3—C4—C5	179.25 (17)	C8—C3—C4—C5	0.4 (3)
C2—C3—C8—C7	−178.98 (16)	C8—C7—C6—C5	0.1 (4)
C2—C3—C8—C9	−0.1 (2)	C8—C9—C10—C11	0.3 (3)
C1—C2—C3—C4	1.4 (2)	C7—C8—C9—C10	178.9 (2)
C1—C2—C3—C8	−179.74 (15)	C9—C8—C7—C6	−179.1 (2)
C3—C2—C1—O1	42.0 (2)	C10—C11—C2—C1	−179.83 (17)
C3—C2—C1—N1	−138.82 (17)	C10—C11—C2—C3	0.7 (3)
C3—C4—C5—C6	−0.5 (3)		

### Naphthalene-1-carboxamide Hydrogen-bond geometry (Å, º)

**Table:**

*D*—H···*A*	*D*—H	H···*A*	*D*···*A*	*D*—H···*A*
C4—H4···O1	0.93	2.38	2.969 (3)	121
N1—H1*A*···O1^i^	0.86	2.10	2.951 (3)	170
N1—H1*B*···O1^ii^	0.86	2.06	2.889 (3)	163

Symmetry codes: (i) −*x*+2, −*y*+1, −*z*; (ii) *x*, −*y*+1/2, *z*−1/2.
